# Adult death registration in Matlab, rural Bangladesh: completeness, correlates, and obstacles

**DOI:** 10.1186/s41118-021-00125-7

**Published:** 2021-07-22

**Authors:** M. Moinuddin Haider, Nurul Alam, Mamun Ibn Bashar, Stéphane Helleringer

**Affiliations:** 1 Health Systems and Population Studies Division (HSPSD), International Centre for Diarrhoeal Disease Research, Bangladesh (icddr,b), Dhaka, Bangladesh; 2 Division of Social Science, New York University Abu Dhabi (NYUAD), Abu Dhabi, United Arab Emirates

**Keywords:** Civil registration and vital statistics, Death registration, Barriers, Enablers, Low- and lower-middle-income countries, Mortality, Survey data, Bangladesh

## Abstract

Civil registration of vital events such as deaths and births is a key part of the process of securing rights and benefits for individuals worldwide. It also enables the production of vital statistics for local planning of social services. In many low- and lower-middle-income countries, however, civil registration and vital statistics (CRVS) systems do not adequately register significant numbers of births and, especially, deaths. In this study, we aim to estimate the completeness of adult death registration (for age 15 and older) in the Matlab health and demographic surveillance system (HDSS) area in Bangladesh and to identify reasons for (not) registering deaths in the national CRVS system. We conducted a sample survey of 2538 households and recorded 571 adult deaths that had occurred in the 3 years preceding the survey. Only 17% of these deaths were registered in the national CRVS system, with large gender differences in registration rates (male = 26% vs. female = 5%). Respondents who reported that a recent death in the household was registered indicated that the primary reasons for registration were to secure an inheritance and to access social services. The main reasons cited for not registering a death were lack of knowledge about CRVS and not perceiving the benefits of death registration. Information campaigns to raise awareness of death registration, as well as stronger incentives to register deaths, may be needed to improve the completeness of death registration in Bangladesh.

**Supplementary Information:**

The online version contains supplementary material available at 10.1186/s41118-021-00125-7.

## Background

Civil registration is an administrative process of continuous recording of vital events: births, deaths, and marriages. In many countries following a death, for example, family members of the deceased are required by law to visit an administrative office to report the death and provide information about it. This includes the particulars of the deceased (such as his or her name) as well as demographic information about the person. If a medical death certificate is available, information about the cause of death might also be recorded. Registering the death benefits families: in addition to complying with state laws, death registration might help them secure an inheritance and access financial and social services that a person or family may be eligible to receive after the death of a relative.

A well-functioning civil registration and vital statistics (CRVS) system also allow for the production of timely age-, sex-, and cause-specific mortality statistics (AbouZahr, De Savigny, Mikkelsen, Setel, Lozano, & Lopez, 2015). CRVS systems provide numerators for important indicators of progress toward the Sustainable Development Goals, such as the neonatal mortality rate and maternal mortality ratio (United Nations Statistics Division, 2016). These data then help governments formulate policies, implement population health programs, and evaluate the effects of these actions. Thus, complete and accurate civil registration of deaths (and births) may improve population health (Lima & Queiroz, 2014; Phillips et al., 2015). Accurate counts of deaths generated by a functioning CRVS system are also essential to monitor the effects of epidemics and other disasters. Such data are thus extensively used in high-income and some upper-middle-income countries to measure monthly or weekly levels of excess mortality during the COVID-19 pandemic (Aburto et al., 2021; Economic Commission for Latin America and the Caribbean (ECLAC)., 2021).

One indicator that is used to track progress toward achieving the Sustainable Development Goals concerns revitalizing global partnerships for CRVS. This approach seeks to increase the number of countries with 100% birth registration and 80% death registration (goal 17; target 19; indicator 2b) (United Nations, 2021). Achieving this objective is challenging in low- and lower-middle-income countries in Asia, Africa, and South America that have deficient CRVS systems (AbouZahr, De Savigny, Mikkelsen, Setel, Lozano, Nichols, et al., 2015; Setel et al., 2007; United Nations Statistics Division, 2020). In these countries, death registration remains largely incomplete (United Nations Statistics Division, 2020; World Health Organization, 2014). Deaths are also often registered with delays, thus precluding the timely monitoring of mortality trends.

There are multiple barriers to death registration in low- and lower-middle-income countries. Lack of knowledge of the CRVS process and its benefits have been identified as the major barriers to the civil registration of deaths of children under age five in a large city in Guinea-Bissau (Fisker et al., 2019). In Nigeria, interrupted power supply, inadequate information, and communication technology infrastructure, and lack of trained personnel appeared as crucial systemic barriers in multiple studies (Maduekwe et al., 2017; Makinde et al., 2020). A recent study in India explored limited time availability, financial hardship, and limited knowledge of CRVS among relatives of recently deceased individuals as possible reasons for delays in death registration (Anupamdeep et al., 2019). In addition, cultural beliefs and practices, logistical and transportation constraints, and perceived low quality of services were found to be reasons for delays in birth registration in Indonesia (Bennouna et al., 2016). These issues may also affect the timeliness of death registration.

### Context of civil registration in Bangladesh

Bangladesh is a lower-middle-income country in South Asia with a population of 165 million. Every year, 3 million births and 0.9 million deaths are estimated to occur in the country (United Nations Department of Economic and Social Affairs, 2019). To understand the dynamics of mortality and fertility, the country mainly depends on censuses, surveys, and a sample registration system (Ahsan et al., 2017). The colonial government of British India established a birth, death, and marriage registration system that was promulgated during the late nineteenth century. This system remained in operation for over a century, but achieved only very low levels of event registration (Local Government Division, 2014). Due to the importance of timely data on births and deaths, the government of Bangladesh repealed this registration system and enacted the Birth and Death Registration Act, 2004 to establish a new functional CRVS system (Local Government Division, 2014). The 2004 act has been amended multiple times since then, such as in 2006. The current CRVS system follows the most recent amendment, adopted in 2018 and published in The Bangladesh Gazette [SRO No. 79-Act/2018] (Ministry of Local Government, 2018). A brief overview of death registration procedures is provided in Table 1.

**Table 1:** A﻿ brief overview of death registration procedures in Bangladesh

*Birth and Death Registration Act*	In 2004, the Bangladesh government repealed the Birth, Death and Marriage Registration Act, 1886 and enacted the Birth and Death Registration Act, 2004.
*Time requirement for death registration*	45 days
*Charge for death registration*	Free within 45 days
*Late registration fee*	Less than $1 USD
*Responsibility to report event for registration*	Primarily parents, children, siblings, and other relatives. Any other can also report.
*Information required for death registration*	Name, sex, age, present and permanent address of the deceased; his/her father’s and mother’s name; husband’s or wife’s name (if married); place of death; and cause of death (according to the International Classification of Diseases [ICD]).
*Documents required for death registration*	Deceased national identity card; birth certificate; certificate/proof of death from health center/provider, graveyard authority, funeral facilitator, or administrator of local government; or post-mortem report; or any other document that proves the death.
*Online registration platform*	Yes
*Enforcement of law*	Limited

Note on causes of death: More than half of the deaths occur at home. Among the deaths that occur at home, the causes of deaths mostly remain unknown. Assigned causes of deaths for deaths that occur at health facilities are often questionable and are not properly aligned with ICD codes (Hazard et al., 2017)

Source: Bangladesh’s birth and death registration rule 2018 (Ministry of Local Government, 2018)

Since 2010, Bangladesh has deployed an online registration system that can be accessed at registration offices under the guidance of administrative workers (deaths cannot be registered online without visiting a registration office). At present, 124 local offices of 11 city corporations, 320 city councils in suburban areas, 4571 union councils in rural areas, and 15 cantonment boards actively register vital events, including deaths. Death registration can also be done in the 55 embassies abroad, mainly located in countries with a high density of Bangladeshi nationals (Local Government Division, 2014).

Bangladesh, along with other Asian countries participating in the Ministerial Conference on CRVS in Asia and Pacific, proclaimed 2015–24 “the Asian and Pacific CRVS Decade” and committed to “Get everyone in the picture” through CRVS (United Nations Economic and Social Commission for Asia and the Pacific, 2015). In 2017, a national survey of women of reproductive age asked mothers to report whether the births of their children under age five were registered. This was the case for only 25% of under-five children (ICF, 2012). No similar estimates based on direct survey reports are available for death registration. Indirectly, however, it is apparent that death registration is incomplete. For example, only 99,871 deaths were registered in 2015 (Local Government Division, 2016), whereas models and projections estimate that 0.9 million deaths likely occurred in the country that year (United Nations Department of Economic and Social Affairs, 2019). This suggests that only approximately 1 in 10 deaths might have been registered in 2015 in Bangladesh.

In this study, we aim to estimate the completeness and correlates of death registration in rural Matlab, Bangladesh. We also explore potential barriers and enablers to death registration as reported by family members of recently deceased adults.

## Methods

### Data sources

This paper is a part of the adult mortality estimation and validation (AMEV) study. The primary goal of this study was to compare survey data on adult mortality generated by siblings’ survival histories to records of deaths collected by the Matlab Health and Demographic Surveillance System (HDSS) area﻿ (Alam et al., 2017). In doing so, the study relied on a study design previously used in Matlab﻿, as well as in Guinea-Bissau, and Senegal, to assess the quality of survey data on mortality (Helleringer et al., 2015; Helleringer et al., 2020; Masquelier et al., 2020); Shahidullah, 1995. Measuring the completeness of death registration among the population of the Matlab HDSS was an exploratory goal of the AMEV study.

The Matlab HDSS has been operated by the International Centre for Diarrhoeal Disease and Research, Bangladesh (icddr,b) since 1966. It conducts household visits every 2 to 3 months to record births, deaths, migrations, marriages, divorces, and selected reproductive and child health information from around 60,000 households. For each recorded death, the HDSS conducts a verbal autopsy that is a comprehensive questionnaire focused on the circumstances and symptoms prior to the death. Using this information, causes of deaths are attributed after review by a trained clinical officer.

HDSS households are distributed in 142 villages over 184 km^2^. Matlab HDSS does not conduct campaigns or programs promoting knowledge and practice of civil registration, including death registration. Since 2016, the HDSS ascertains whether death is registered at the time of the verbal autopsy. However, most verbal autopsies are conducted within a short time after the death (such as a few weeks), frequently before the legal day for death registration has passed (i.e., 45 days; see Table 1). Since there is no follow-up after the initial verbal autopsy and late death registrations (more than 45 days after death) are common in Bangladesh, HDSS data underestimate the completeness of death registration in the area. Nonetheless, some of the respondents in the AMEV study might have previously been asked about death registration if they served as a verbal autopsy informant after the death of their relative(s). In that case, their level of awareness about CRVS might be slightly higher than HDSS residents who did not participate in a verbal autopsy interview or residents of neighboring areas not covered by the HDSS. More details about the Matlab HDSS operations and quality of collected data are available elsewhere (Alam et al., 2017).

For the AMEV study, we drew a stratified random sample of households of the Matlab HDSS area. We used information on individual household members, their family ties, and the time since a family member’s death to form sampling strata. Specifically, we oversampled households with the death of an adult (age 15 years and above) over the past 3 years. We also oversampled households where a sibling of a recently deceased adult resided. We thus formed three strata: (1) households with a member who is a sibling of a recently deceased adult, (2) other households with an adult death in the past 3 years, and (3) all other households. The first sampling stratum was further subdivided according to the cause and timing of the sibling’s death. This sampling scheme was adopted to ensure sufficient numbers of reported deaths in the survey data collected as part of the AMEV study, similar to procedures used in previous validation studies of survey data on mortality (Helleringer et al., 2014; Shahidullah, 1995).

Some households thus had *target respondents*, in other words, the household member whose brother(s) or sister(s) had recently died. If the target respondent had moved to another household, including outside of the HDSS area, that new household was added to the list of study households. In other households, the household informant could be any adult older than 18 years. In all households, there was at most one household informant. Sample size calculations indicated that approximately 2400 households were required to meet the primary objectives of the AMEV study. Eventually, we recruited 2538 households, in which 626 deaths of household members were reported to have occurred since January 2016.

### Data collection activities

Data collection for the AMEV study occurred from July 2019 to March 2020. Interviewers were provided with a list of households to survey. They located these households using information contained in the HDSS datasets, such as village of residence and name of the household head. Each household informant was asked to complete a questionnaire, which was adapted from household questionnaires used during national censuses and the demographic and health surveys program (Corsi et al., 2012). Respondents were first asked to list all household members and to report some of their characteristics, such as age, educational level, and relationship to the household head. Then they were asked to list all deaths of household members that had occurred since January 2016 (i.e., 3.5 to 4 years prior to the survey, depending on the timing of data collection).

For each reported death, we asked respondents to state the circumstances of the death, including the place of death and whether the death was due to violence or to an accident. For deaths of women ages 15 to 49, we also asked whether the death occurred during pregnancy, at the time of childbirth, or within 42 days of the birth. Finally, we asked informants whether the death had been registered with the national civil registration system and what the reasons were for registering or not registering the death (Additional file 1).

To assess the registration of deaths in the national CRVS system, we asked household respondents whether they obtained a death certificate for the deceased from administrative authorities such as the union council (in rural areas), city council (in urban areas), or city corporation. This made it possible to avoid potential confusion with other documents that might be issued around the time of death but do not constitute death registration, such as a medical death certificate, or a burial permit. A *medical death certificate* is a document stating the deceased’s age, sex, time of death, and cause of death. It is usually issued by a medical practitioner when the person dies at a health facility. The *burial permit* is issued primarily by city corporations to allow access to cemetery grounds for a dead body. In Bangladesh, some administrative authorities also issue *succession certificates*, which list the names of the successors of property of the deceased and their relations with him or her. Issuing a succession certificate requires registration of the death in the national civil registration system. However, issuing a medical death certificate does not require prior registration of the death in the national civil registration system.

Respondents who reported registering the death were not asked to show the death certificate or any other documents to study interviewers. The list of reasons for (not) registering deaths was adapted from prior studies conducted in Indonesia and Guinea-Bissau (Duff et al., 2016; Fisker et al., 2019). We then refined the list during the AMEV study’s field pilot and interviewers’ training and practice. Respondents were allowed to list multiple reasons for (not) registering a death. Interviewers did not read the list of possible reasons to study participants. After each reason reported by respondents, interviewers were instructed to probe non-specifically by asking, “Was there another reason why you did (not) register the death?” If a reason mentioned by a respondent did not appear in the list of possible reasons, interviewers could record it in a follow-up field.

The questionnaires were translated into Bengali, the local language of the study area. Study interviewers were native Bengali speakers and were familiar with the local dialect to facilitate effective communication with respondents. The data collection team went through extensive training on questionnaires, field practice before the survey, and frequent debriefing sessions on field observations related to data collection during the survey.

### Data analysis

During the AMEV study, 626 deaths were reported by household informants. Of these deaths, 30 were reported in households of target respondents who had moved outside of the Matlab HDSS area and were excluded. An additional 25 deaths occurred at ages below 15 years and were not included. Analyses of death registration were thus conducted using the remaining 571 deaths. Due to oversampling of some population groups in the AMEV study, the age and sex distribution of the deaths reported during the survey differs from the deaths recorded by the HDSS in the Matlab area. We thus calculated sampling weights so that the age and sex distribution of the deaths captured in the survey matches the age and sex distribution of deaths in the population of the Matlab HDSS. Details of the calculations are shown in Additional file 2.

We first described the characteristics of the respondents who completed the household questionnaire on residents and deaths, along with the socio-demographic characteristics of the deceased. Then we estimated the completeness of death registration as the (weighted) percentage of deaths that were reported as registered to the national CRVS system. We also linked the AMEV survey data to HDSS data so we could obtain additional information on the relative poverty of the deceased. This characteristic was measured using a household wealth index, computed using socio-economic data collected by the HDSS in December 2014 (icddr,b, 2016). We then measured the frequency of various reported reasons for (not) registering deaths to the national CRVS system by the sex of the deceased.

Finally, to examine the association between death registration and several socio-demographic variables, we used logistic regression models. In these models, the outcome variable is binary: it takes value 1 when the death is reported as registered to the national CRVS system, and it takes value 0 otherwise. Covariates included sex (male, female), age at death (15–34, 35–44, 45–54, 55–64, 75–84, 85+ years), wealth quintile of the household (lowest, second, middle, fourth, highest, missing), cause of death (whether the death was due to an accident/injury), place of death (at a health facility or outside a health facility), and year of death.

We considered two specifications of the logistic regression model: one model with socio-demographic covariates only, and another model that included village fixed effects alongside socio-demographic covariates. The inclusion of village fixed effects was motivated by potential unobserved heterogeneity between villages that might confound the relationship between covariates and registration outcomes. For example, villages closer to CRVS offices may have higher death registration rates than other villages. The 571 deaths analyzed in this paper were recorded in 528 households: in 488 households, only one death was reported, whereas 37 households reported two deaths each, and three household informants reported three deaths in their household. In all models, we thus adjusted standard errors for the clustering of observations within households. We reported crude odds ratios as well as regression-based adjusted odds ratios associated with socio-demographic covariates.

## Results

More than 80% of respondents who completed the household questionnaire were women. Most household informants were 25 to 54 years old, with only 8% of household informants aged 55 years and older and 11% aged 18 to 24 years. Around 96% of respondents had been married; 81% of respondents were married at the time of the AMEV study. Only 12% of respondents had never attended school, and more than half had achieved higher than primary education (Table 2).

**Table 2:** Characteristics of respondents to recent household deaths modules, Adult mortality estimation and validation survey, 2019–2020

Characteristics	Percent	Number (unweighted)
Total	100.0	2538
Age of respondents		
18–24	10.6	270
25–34	23.2	589
35–44	38.0	964
45–54	20.1	509
55–64	5.9	149
65–74	1.7	44
75+	0.5	13
Sex of respondents		
Male	19.5	496
Female	80.5	2042
Marital status of respondents		
Never married	3.7	95
Currently married	81.3	2062
Widowed/divorced/separated/deserted	15.0	381
Education of respondents		
Never attended school	12.1	307
Primary	36.0	914
Secondary incomplete	32.0	813
Secondary complete	6.0	153
Above secondary	13.8	351
Total household members		
1	3.9	99
2	7.8	198
3	18.5	469
4	25.1	638
5+	44.7	1134

Table 3 shows the socio-demographic characteristics of the deceased as well as the circumstances of reported deaths. More than half of the deaths reported to have occurred in study households since 2016 were males. Female deaths occurred at older ages than male deaths; 56% of female deaths occurred at more than 75 years, as opposed to 31% of male deaths. A smaller proportion of deaths occurred among higher wealth quintiles. Male deaths were more frequently due to accidents or injuries than female deaths (5.1% vs. 1.4%). Fewer than 1 in 5 reported deaths occurred in health facilities.

**Table 3:** Characteristics of deceased adults by sex, Adult mortality estimation and validation survey, 2019–2020

Characteristics	Weighted percentage			Weighted number			Unweighted number		
	Male	Female	Both	Male	Female	Both	Male	Female	Both
Sex distribution^a^	56.0	44.0	100.0	-	-	-	-	-	-
Total	100.0	100.0	100.0	320	251	571	355	216	571
Age at death									
15–34^b^	3.5	4.1	3.7	11	10	21	39	33	72
35–44	3.8	2.8	3.4	12	7	19	43	26	69
45–54	12.3	4.1	8.7	39	10	50	98	27	125
55–64	22.5	11.5	17.6	72	29	101	90	36	126
65–74	27.1	22.1	24.9	87	55	142	37	24	61
75–84	23.0	36.3	28.8	74	91	165	27	30	57
≥ 85	7.9	19.2	12.9	25	48	73	21	40	61
Wealth quintile									
Lowest	19.4	18.3	18.9	62	46	108	79	43	122
Second	21.3	16.6	19.3	68	42	110	64	44	108
Middle	15.7	20.9	18.0	50	52	103	64	41	105
Fourth	24.5	15.9	20.7	78	40	118	72	31	103
Highest	12.3	17.2	14.5	39	43	83	51	34	85
Missing	6.8	11.1	8.7	22	28	50	25	23	48
Cause of death									
Injuries/accident	5.1	1.4	3.5	16	3	20	29	6	35
Other natural	94.9	98.6	96.5	304	247	551	326	210	536
Place of death									
Facility	21.9	13.9	18.4	70	35	105	112	48	160
Out of facility	78.1	86.1	81.6	250	216	466	243	168	411
Year of death									
2016	27.1	26.9	27.0	87	67	154	88	56	144
2017	26.0	25.6	25.8	83	64	148	102	59	161
2018	30.1	24.6	27.7	96	62	158	115	55	170
2019^b^	16.7	22.9	19.4	54	57	111	50	46	96

^a^10-year age groups 15–24 and 25–34 are collapsed into one due to very small numbers

^b^Deaths in 2019 include one death in early 2020

The proportion of reported deaths that were registered was low at 17% (Fig. 1). We found a very large gender gap in death registration: only 5% of female deaths were registered, compared to 26% of male deaths (Fig. 1). Completeness of death registration varied across age groups from 18 to 34% for males and 2 to 20% for females: the highest rates were in the 45- to 54-year-old age groups for both males (34%) and females (20%). Death registration was the lowest in the poorest wealth quintiles. For male deaths, death registration appeared to be varied over time, from 17% of deaths in 2016 to 33% of deaths in 2019. We did not find a similar pattern for female deaths. Registration rates were related to the cause of death, with deaths due to *injuries and accidents* registered at a much higher rate than deaths from *natural causes* among males (25.0% vs. 38.3%). There were too few deaths due to accidents and injuries among women to reliably measure registration rates by cause among that population group. Finally, registration rates varied according to the place of death, particularly among women: whereas 16.5% of the deaths that occurred in health facilities were registered, this was the case for only 3.3% of the deaths that occurred outside of health facilities (such as at home). We did not find a similarly large gap among male deaths.

**Fig﻿. 1:**
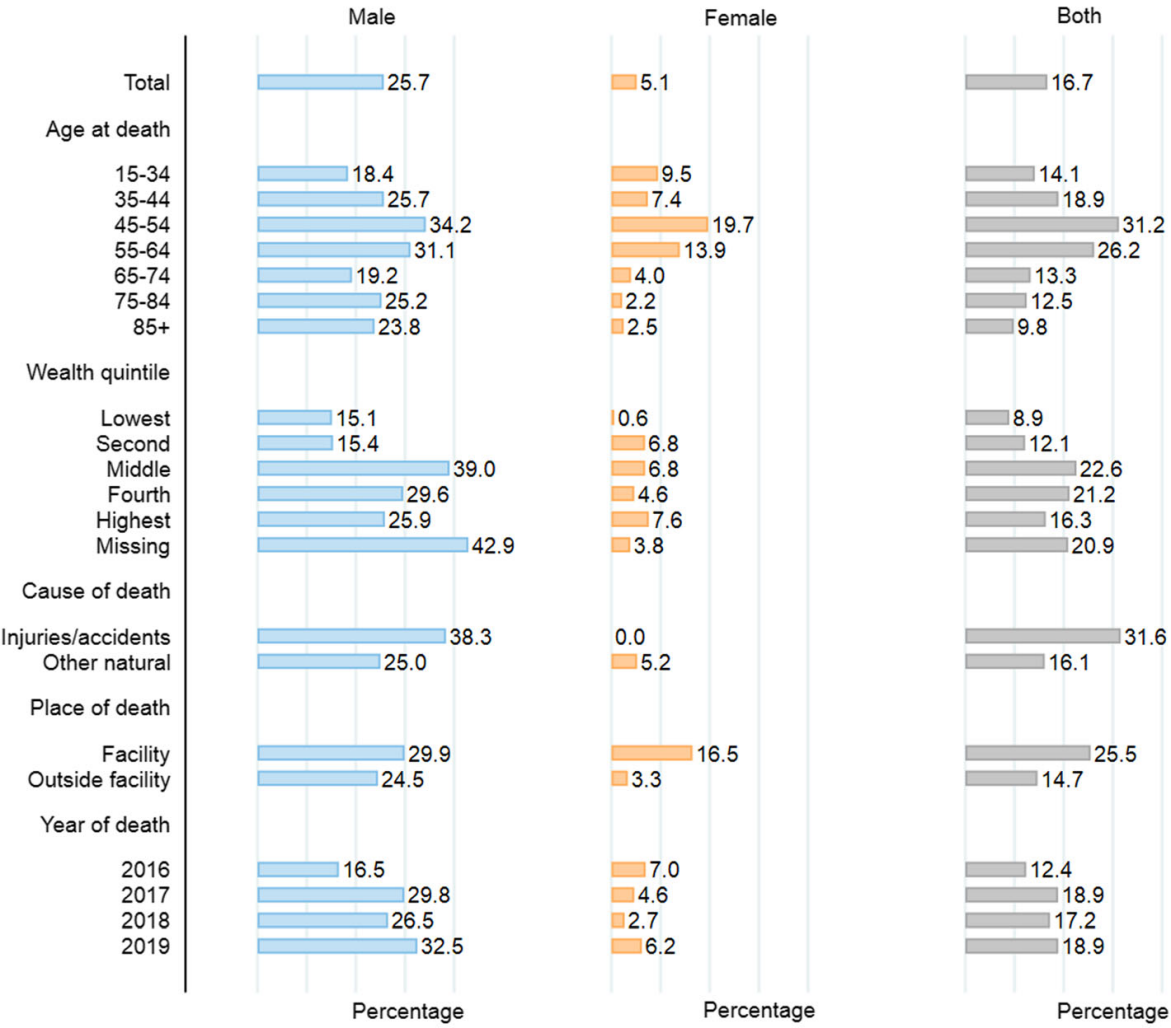
Completeness (%) of death registration by deceased’s characteristics, adult mortality estimation and validation survey, 2019–2020

The results of the regression model in Table 4 revealed no significant difference in odds ratios between age groups, injury/accidental and other deaths, or facility and outside facility deaths, and no significant change over 2016 to 2019. The gender of the deceased remains significant, with the odds of female death registration being around 85% lower than the odds of male death registration. Odds of death registration for deaths in the fourth and the highest wealth quintiles are multiple times higher than deaths in the lowest wealth quintile (Model 2, Table 4). Gender-specific models also found differences in death registration in wealth quintiles and no difference in other covariates (gender-specific models are not shown).

**Table 4:** Crude and adjusted odds ratios of death registration, Adult mortality estimation and validation survey, 2019–2020

Death registration to CRVS	cOR	Model 1 Adjusted for correlation of observations within households			Model 2^a^ Adjusted for correlation of observations within households and village effects		
		aOR	95% CI		aOR	95% CI	
			Lower limit	Upper limit		Lower limit	Upper limit
Female (*Ref: Male*)	0.16**	0.16***	0.07	0.36	0.15***	0.06	0.36
Age at death (*Ref: 15–34 years*)							
35–44	1.42	1.32	0.45	3.86	0.85	0.15	4.79
45–54	2.76	2.18	0.80	5.98	3.01	0.75	12.07
55–64	2.16	1.86	0.68	5.12	1.62	0.39	6.69
65–74	0.93	1.07	0.32	3.55	0.57	0.12	6.63
75–84	0.87	1.07	0.29	3.90	0.33	0.07	1.57
85+	0.66	0.99	0.27	3.59	.45	0.09	2.28
Wealth quintile (*Ref: Lowest*)							
Second	1.41	1.52	0.57	4.07	2.97	0.75	11.70
Middle	2.98**	1.86**	1.39	10.75	3.24	0.94	11.13
Fourth	2.74*	2.62	0.95	7.28	10.21**	2.56	40.64
Highest	1.99	2.46	0.96	6.27	5.01*	1.44	17.48
Missing	2.69*	3.94*	1.03	15.00	8.53**	1.94	37.53
Injuries/accidental deaths (*Ref*: *Yes*)	0.42	0.58	0.17	1.95	0.73	0.20	2.69
Facility death (*Ref*: outside facility death)	0.50**	0.73	0.33	1.61	0.81	0.33	1.99
Year of death	1.14	1.17	0.85	1.62	1.32	0.96	1.81
Constant	-	0.17^*^	0.03^*^	0.85	0.04^*^	< 0.01	0.77
Number	571	571	-	-	379	-	-

**p < 0.01; *p < 0.05; cOR stands for crude odds ratio; aOR stands for adjusted odds ratio

^a^Model 2 excludes villages having all deaths registered or no deaths registered; thus, drops 192 observations

Figure 2 shows that the most commonly reported reason for registering a death was to obtain documents for inheritance (48 to 55%), followed by enabling access to social, financial, and legal services, such as pensions, that the relatives of a deceased individual might be eligible to receive. The most common reason for not registering a death was unawareness of the importance of death registration (72%), followed by not knowing about death registration at all (26 to 32%).

**Fig﻿. 2:**
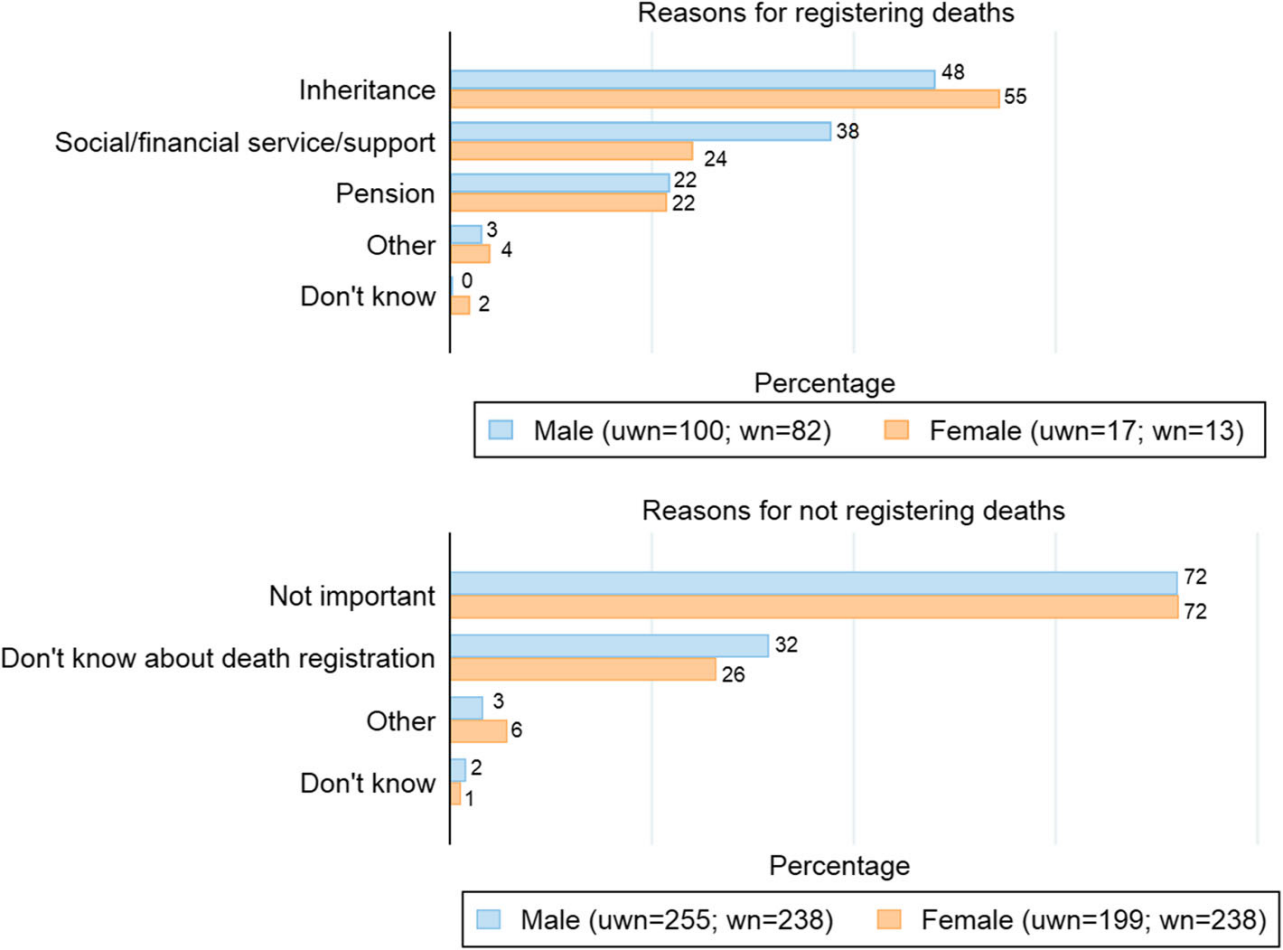
Reported reasons (%) for (not) registering adult deaths, Adult mortality estimation and validation survey, 2019–2020. Notes: Legends—*uwn*=unweighted number and *wn*=weighted number. Due to multiple answers, sum of the percentages may exceed 100. Specific reasons within broad categories of reasons of (not) registering deaths are shown in Additional file 3

## Discussion

Our study has documented the limited completeness of death registration (17%) in the Matlab area of Bangladesh. This rate is comparable to the rate that can be inferred from the number of registrations compiled by the national CRVS systems (Local Government Division, 2016). Our study has also highlighted large gender differences in registration rates among adult deaths (those over 15 years of age) as well as disparities in registration related to age and socio-economic status. Deaths in the poorest households in particular were less likely to be registered than deaths that had occurred in more affluent households.

Our study helps increases understanding of why the death registration rate remains low in this area of Bangladesh. On the one hand, a large proportion of the household informants we interviewed stated lack of knowledge about CRVS as a key reason for not registering a recent household death. Others cited not perceiving any benefits associated with death registration. These knowledge-related barriers are similar to barriers to birth and death registration that limit the coverage of CRVS in other low- and lower-middle-income countries (Fisker et al., 2019). On the other hand, among the minority of informants who reported a registered death, the primary reasons cited for registration were related to inheritance and access to social services and benefits. However, such reasons might provide incentives to register only a minority of the adult deaths that occur in this area of Bangladesh. A small proportion of men in rural areas works in the formal sector, whereas the rest work in the informal sector, in activities such as farming, trading, or house building, which seldom provide access to social benefits. There are social/financial support programs like the Vulnerable Group Development Program or Vulnerable Group Feeding Program for destitute men and women from poor or ultra-poor households (Begum, 2018). However, the coverage of such social safety net programs is below 30% (Bangladesh Bureau of Statistics, 2017). So, few surviving relatives may have a need to register the death of their relative(s) to secure Vulnerable Group Development or Vulnerable Group Feeding Program support.

The reasons stated by respondents for registering deaths also help us understand the emergence of a gender gap in death registration. For example, when a death was registered, securing rights to inherit properties of the deceased was the reason most commonly reported (males: 48%; females: 55%). Since only 13% of women own agricultural land (solely or jointly), compared to 70% of men (Solotaroff et al., 2019), this motivation might explain why male deaths are registered at a higher rate than female deaths. Respondents also stated more frequently that deaths of male relatives were registered to obtain social safety benefits (male deaths: 38%; female deaths: 24%). This might be the case because men are more likely to work in the formal sector in Bangladesh than are women.

The limited coverage of death registration, particularly among women, might also be related to the fact that the large majority of deaths occurs outside of health facilities in this area of Bangladesh. Even though the association of place of death with registration outcomes disappeared in multivariate models, we found very low crude rates of registration among deaths that occurred at home. Increasing registration rates among such deaths might require community-based interventions and the deployment of recording systems that reach villages and remote areas. Sri Lanka’s community-based death notification system by *Grama Niladhari* (village officer) provides an example of a system that facilitates high levels of death registration completeness (Sri Lanka Implementation Working Group, 2018).

In Bangladesh, the CRVS legislation also suggests that field staff, such as health assistants and family welfare assistants, of the Ministry of Health and Family Welfare distribute death registration application forms to the family of the deceased during field visits, complete the forms with the help of the deceased’s household members, and return the completed forms to the CRVS office (Ministry of Local Government, 2018). Then the CRVS office issues the death certificates and gives them to the health assistants and/or family welfare assistants, who then give them to the deceased’s household or family (Ministry of Local Government, 2018). A recent pilot study in rural Bangladesh has shown that involving health assistants and family welfare assistants improved birth and death notification (Tahsina et al., 2020).

Even among the deaths that occurred in health facilities (approximately 1 in 5 deaths), registration rates remained low (less than 33%). This constitutes a missed opportunity due to the lack of an efficient death notification system from health facilities to CRVS offices. Several Asian and African low- and lower-middle-income countries have initiated health facility–based birth and death notification systems to inform the CRVS system of the occurrence of events (Kasasa et al., 2020; Sri Lanka Implementation Working Group, 2018). This makes it possible to register not only births and deaths at facilities, but also stillbirths at facilities (Kasasa et al., 2020).

### Limitations and implications

There are several limitations to this study. First, respondents were not asked to show the death registration certificate to the interviewers if they reported that the death had been registered. As a result, our figures might overestimate the coverage of death registration. This might be the case, for example, if a respondent confuses death registration with another administrative or medical process that occurs at the time of death and generates paperwork. It might also occur due to social desirability bias, that is, respondents providing interviewers with answers that are deemed more acceptable or socially valued. Second, the available sample sizes were too limited to investigate several aspects of death registration. For example, due to the small number of registered female deaths reported in our sample, we were not able to precisely characterize the reasons for registering such events. Larger studies would allow for further investigation of such patterns and for the formulation of gender-specific strategies to encourage death registration. Third, some household informants might not be aware of the registration status of a death. Fourth, we did not investigate the timeliness of death registration in our questionnaire. In Bangladesh, as in many other countries, death registration must occur within a certain timeframe after the death. This ensures that this information can be aggregated into vital statistics in a timely manner, so as to monitor mortality trends in (near) real time. Assessing the proportion of events that are reported within that timeframe is thus an important component of the evaluation of CRVS systems. Future studies should therefore inquire about the date of registration relative to the date of death. Fifth, our study excluded a number of events that occurred in households of individuals who had migrated out of the Matlab HDSS area. If registration rates are different in such households, our estimates of completeness might be affected. Finally, our study was nested within the activities of the Matlab HDSS, a long-standing demographic data collection system. Due to repeated household visits by fieldworkers, local residents might have a higher awareness of civil registration than residents of neighboring areas not covered by the Matlab HDSS. Our results thus may not be representative of other parts of Bangladesh.

Despite these limitations, our study has several important implications. As in other low- and lower-middle-income country settings, information and awareness campaigns about death registration and its benefits are needed. Such campaigns might help address key barriers that keep registration low. Since only a small fraction of the families who have experienced a death might have a need to secure inheritance documents or social benefits, additional incentives to register deaths might be needed. A recent study in Agincourt, South Africa, pointed to the usefulness of financial incentives in improving the completeness of vital events in the area (Garenne et al., 2016). Similarly, community-based outreach activities might be needed to connect rural families with the administrative systems that oversee death registration. This is especially the case in Bangladesh, where more than 80% of deaths occur at home. The Ministry of Local Government has community-based elected representatives who often participate in funeral prayers and interact with local religious leaders and bereaved family members. Such local actors might help raise awareness of death registration and mobilize communities toward global registration objectives.

## Conclusion

Civil registration of deaths is very low in rural Bangladesh, particularly among women and members of poorer households. Securing inheritance and gaining access to social support services and pensions are the two predominant reasons for death registration. On the other hand, limited knowledge of the death registration system and of the importance and benefits associated with death registration are the most commonly reported reasons for not registering a death. Raising community awareness about death registration, such as through information campaigns, might contribute to the completeness of death registration. Additional incentives to register deaths are also needed to ensure that the global objective of 80% death registration is reached in this area of Bangladesh (United Nations, 2021).

## Supplementary Information

**Additional file 1.** Questions related to death registration and list of possible answers.

**Additional file 2.** Age-specific deaths in the population and sample, and calculated weights to be used in sample data analysis.

**Additional file 3.** Reasons for (not) registering deaths.

## Data Availability

The datasets used during the current study are available from the corresponding author upon reasonable request.

## Abbreviations

AMEV: Adult mortality estimation and validation
CRVS: Civil registration and vital statistics
HDSS: Health and demographic surveillance system
icddr,b: International Centre for Diarrhoeal Disease Research, Bangladesh
