# Supplementary material for: Adult death registration in Matlab, rural Bangladesh: completeness, correlates, and obstacles
Source: Genus. 2021 Jul 22;77(1):13. doi: 10.1186/s41118-021-00125-7 (PMC8295546; doi:10.1186/s41118-021-00125-7)
Supplement: Supplementary file 1 — Additional file 1. Questions related to death registration and list of possible answers. [file 41118_2021_125_MOESM1_ESM.docx]

Additional file 1: Questions related to death registration and list of possible answers

| **Sl. #** | **Question** | **Answer options** |
| --- | --- | --- |
| Q39 | Was a death certificate established after the death of (NAME)?  ***Instructions for interviewer:*** *If ‘yes,’ ask whether they collected the certificate from any doctor, health facility, union council or city council or city corporation. If the certificate was collected from ‘union council or city council or city corporation,’ then circle ‘yes.’* | Yes 1  No 2🡪go to Q41 |
| Q40 | Why was the death of (NAME) registered in the civil registry?  ***Instructions for interviewer:*** *Do not read out the list of reasons. Let the respondent spontaneously report. After reporting of each reason, ask them, “Was there another reason why you registered the death?”*  *Multiple answers allowed* | So government can count deaths A  For inheritance B  To access social services C  To obtain pension D  To obtain burial permit E  To remember [NAME] F  Other (specify) X  Don’t know Z |
| Q41 | Why was the death of (NAME) not registered in the civil registry?  ***Instructions for interviewer:*** *Do not read out the list of reasons. Let the respondent spontaneously report. After reporting of each reason, ask them, “Was there another reason why you did not register the death?”*  *Multiple answers allowed* | Don’t know what death registration is A  Don’t know how to register a death B  Process too costly C  Place of registration too far D  Registration process too complicated E  Don’t have the required documents F  Not important to register a death G  Other (specify) X  Don’t know Z |
| Note: Reasons listed in Q40 and Q41 are adopted from studies in Indonesia and Guinea-Bissau | | |
|  | | |
