# Supplementary material for: Adult death registration in Matlab, rural Bangladesh: completeness, correlates, and obstacles
Source: Genus. 2021 Jul 22;77(1):13. doi: 10.1186/s41118-021-00125-7 (PMC8295546; doi:10.1186/s41118-021-00125-7)
Supplement: Supplementary file 2 — Additional file 2. Age-specific deaths in the population and sample, and calculated weights to be used in sample data analysis. [file 41118_2021_125_MOESM2_ESM.docx]

Additional file 2. Age-specific deaths in the population and sample, and calculated weights to be used in sample data analysis

| Deaths in Matlab HDSS (2016–2019) | | | Deaths in survey | Weights |
| --- | --- | --- | --- | --- |
| Age | Number | Proportion |  |  |
| [1] | [2] | [3] | [4] | [5]=([3] × 571)/[4] |
| 15–19 | 57 | 0.009394 | 23 | 0.233205 |
| 20–24 | 53 | 0.008734 | 19 | 0.26249 |
| 25–29 | 56 | 0.009229 | 17 | 0.309977 |
| 30–34 | 61 | 0.010053 | 13 | 0.441547 |
| 35–39 | 84 | 0.013843 | 30 | 0.263481 |
| 40–44 | 120 | 0.019776 | 39 | 0.289539 |
| 45–49 | 197 | 0.032465 | 72 | 0.257469 |
| 50–54 | 330 | 0.054384 | 53 | 0.585907 |
| 55–59 | 497 | 0.081905 | 59 | 0.792675 |
| 60–64 | 572 | 0.094265 | 67 | 0.803363 |
| 65–69 | 667 | 0.109921 | 25 | 2.510593 |
| 70–74 | 843 | 0.138926 | 36 | 2.203513 |
| 75–79 | 932 | 0.153593 | 18 | 4.872299 |
| 80–84 | 818 | 0.134806 | 39 | 1.973691 |
| 85+ | 781 | 0.128708 | 61 | 1.204791 |
| Total | 6068 | 1.000000 | 571 | – |
