# Supplementary material for: Adult death registration in Matlab, rural Bangladesh: completeness, correlates, and obstacles
Source: Genus. 2021 Jul 22;77(1):13. doi: 10.1186/s41118-021-00125-7 (PMC8295546; doi:10.1186/s41118-021-00125-7)
Supplement: Supplementary file 3 — Additional file 3. Reasons for (not) registering deaths. [file 41118_2021_125_MOESM3_ESM.docx]

Additional file 3. Reasons for (not) registering deaths

| **Broad category of reasons** | **Reasons included in a broad category** |
| --- | --- |
| *Reason for registering a death* | |
| Inheritance | Inheritance of land or other registered properties; money in bank account; insurance |
| Social/financial service/support | Receive loan; waive loan; NGO supports; social safety nets; other social services |
| Pension | Self-explanatory |
| Other | *Legal issues*: to get justice; to get legal documents; to get burial permission; to remember the deceased; other |
| Don’t know | Self- explanatory |
| *Reason for not registering deaths* | |
| Don’t perceive the importance of death registration | Self- explanatory |
| Don’t know about death registration | Self- explanatory |
| Other | Heard about death registration but don’t know how to register; death registration process is costly/complicated; CRVS office is located too far away; don’t have all necessary documents; other |
| Don’t know | Self- explanatory |
